# The living marine resources in the Mediterranean Sea Large Marine Ecosystem

**DOI:** 10.1016/j.envdev.2020.100555

**Published:** 2020-12

**Authors:** Chiara Piroddi, Francesco Colloca, Athanassios C. Tsikliras

**Affiliations:** a European Commission, Joint Research Centre (JRC), Ispra, Italy; b Integrative Marine Ecology Department, Stazione Zoologica Anton Dohrn, Naples, Italy; c Laboratory of Ichthyology, School of Biology, Aristotle University of Thessaloniki, Thessaloniki, Greece

**Keywords:** Food web, Stock status, Catch, Management, Policy, Ecological models

## Abstract

The Mediterranean Large Marine Ecosystem (Med-LME) is a heterogeneous system that, despite its oligotrophic nature, has high diversity of marine species and high rate of endemism, making it one of the world hotspots for marine biodiversity. The basin is also among the most impacted Large Marine Ecosystems in the world due to the combined multiple stressors, such as fishing pressure, habitat loss and degradation, climate change, pollution, eutrophication and the introduction of invasive species. The complexity of Med-LME in its structure/function and dynamics, combined with the socio-political framework of the region make management of its marine resources quite challenging. This contribution aims at highlighting the importance of the Med-LME, with an emphasis on the state of its food web and of its fish/fisheries using modelling tools and national/international reporting. The purpose is to demonstrate the importance of an holistic framework, based on stock assessments and ecosystem based modelling approaches, to be adopted in support of management and conservation measures for the preservation and sustainable use of the Med-LME resources.

## 1 The living marine resources: an overview

**Fig. 1:**
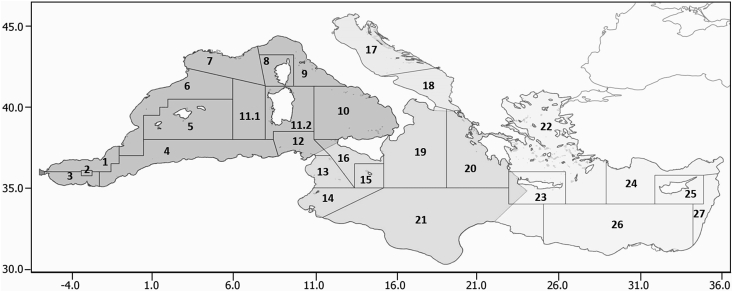
The Mediterranean Sea LME (Med-LME) with the four main divisions accordingly to the European Marine Strategy Framework Directive (MSFD; 2008/56/EC): Western Mediterranean Sea (dark grey); Adriatic Sea (light grey); Ionian and Central Mediterranean Sea (grey); Aegean and Levantine Sea (white), and the twenty seven FAO-GFCM Geographical Sub-Areas (GSAs): Northern Alboran Sea (1); Alboran Island (2); Southern Alboran Sea (3); Algeria (4); Balearic Islands (5); Northern Spain (6); Gulf of Lions (7); Corsica Island (8); Ligurian and North Tyrrhenian Sea (9); South Tyrrhenian Sea (10); Sardinia (west: 11.1); Sardinia (east:11.2); Northern Tunisia (12); Gulf of Hammamet (13); Gulf of Gabes (14); Malta Island (15); South of Sicily (16); Northern Adriatic (17); Southern Adriatic Sea (18); Western Ionian Sea (19); Eastern Ionian Sea (20); Southern Ionian Sea (21); Aegean Sea (22); Crete Island (23); North Levant (24); Cyprus Island (25); South Levant (26); Eastern Levant Sea (27).

The Mediterranean Sea LME (Med-LME) extends from 30°N to 45°N and from 6°W to 36°E and constitutes the largest (2 522 000 km^2^) and deepest (average 1460 m, maximum 5267 m) enclosed sea on Earth (Fig. 1). The basin is oligotrophic with some exceptions along coastal areas mainly due to river discharges (Barale and Gade, 2008) and frontal mesoscale activity (Siokou-Frangou et al., 2010). Biological productivity decreases from north to south and west to east whilst an opposite trend is observed for temperature and salinity (Coll et al., 2010; Costello et al., 2010). Despite its oligotophic characteristic, the Med-LME has a relatively high marine species richness (~17,000 species) and a high rate of endemism, making it one of Earth's hotspot areas for marine biodiversity (Coll et al., 2010; Costello et al., 2010). Of this richness, the majority is represented by the Animalia group (~11,500 species), with the greatest contribution coming from Crustacea (13.2%) and Mollusca (12.4%) (Coll et al., 2010). Among the vertebrates, 650 marine species of fishes (mainly from actinopterygians (86%)), nine species of marine mammals (five Delphinidae, one each for Ziphiidae, Physeteridae, Balaenopteridae, and Phocidae) and three species of sea turtles (the green *Chelonia mydas*, the loggerhead *Caretta caretta* and leatherback *Dermochelys coriacea*) inhabit the Mediterranean Sea. Among the seabirds, 15 species frequently occur in the Mediterranean Sea, 10 gulls and terns (Charadriiformes), four shearwaters and storm petrels (Procellariiformes), and one shag (Pelecaniformes) (Coll et al., 2010).

Concerning the fishery sector, according to the last report of the Food and Agriculture Organization (FAO, 2018a), in the Med-LME, there are approximately 75,000 fishing boats in operation, with small-scale (e.g., gillnet, trammel net boats) accounting for 78% of the total, followed by trawlers (9%), longliners/tuna-seiners/dredges (9%), and purse seiners/pelagic trawlers (4%). Fishing provides around 227,000 direct jobs with reported landings that oscillate to around 800,000 tonnes annually, mostly concentrated in the western Mediterranean and Adriatic Sea, and with a total landing value that is estimated to be more than 3 billion Euros per year. Small pelagics (mainly European anchovy *Engraulis encrasicolus* and European sardine *Sardina pilchardus*) and medium/small sized demersal fish (e.g., European hake *Merluccius merluccius*, red mullet *Mullus barbatus,* Sparidae) comprise the bulk of the total landings (FAO, 2018a).

**Fig. 2:**
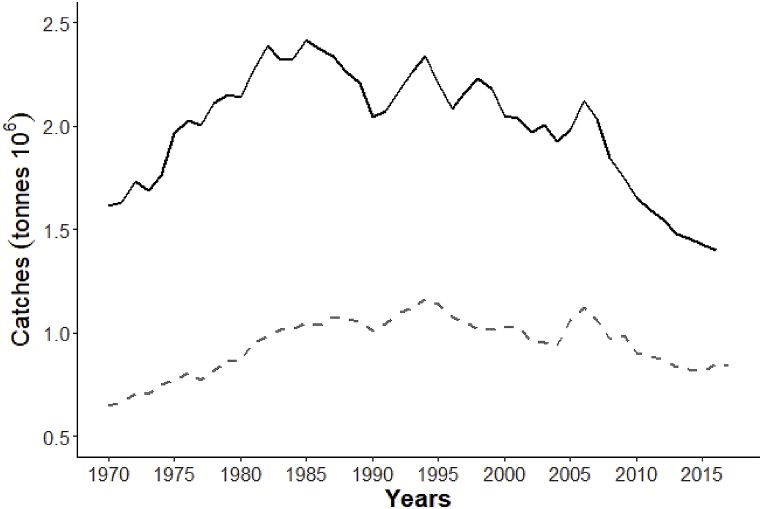
The Mediterranean Sea LME catches reported to FAO (dotted line) and reconstructed catches (black line) for the 1970–2017 period. Reconstructed catches were estimated using a catch-reconstruction approach (Pauly et al., 2014; Pauly and Zeller, 2016) which looked at all types of fisheries removals: from reported and unreported catches (from both industrial and artisanal fisheries) to recreational catch and discards, using official statistics and data from peer-reviewed and grey literature and input of local experts.

The historical trend of reported catches in the Med-LME (1970–2017) (FAO, 2018a) evidences a gradual increase of reported catches that peaked in mid-1990 at around 1.1 million tonnes, followed by a continuous decline afterwards, with the exception of the year 2006 (Fig. 2). Accordingly to recent studies (Pauly et al., 2014; Pauly and Zeller, 2016; Piroddi et al., unpublished), if we incorporate estimates of the unreported catches (e.g., discards, recreational catch) to the reported catches, then the decline is even more pronounced, and with total catches being approximately 2.1 times more than the reported ones (Fig. 2).

### 1.1 A food web perspective

**Fig. 3:**
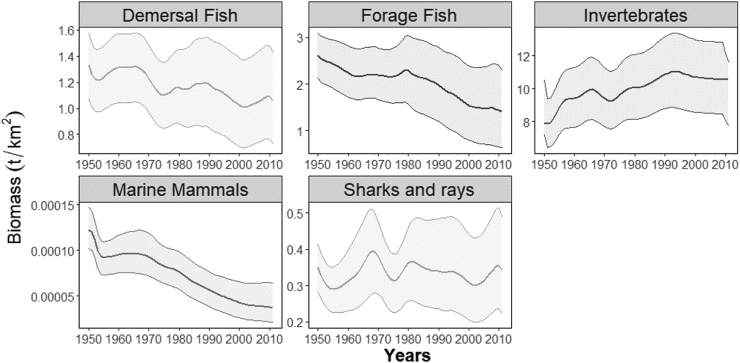
Relative modelled biomass (t/km^2^) for the 1950–2011 period for main functional groups of the Mediterranean Sea LME: Demersal Fish; Forage Fish; Invertebrates; Marine Mammals and Sharks and rays. Shadow represents the 95% percentile and 5% percentile.

In the Mediterranean Sea LME, the major driving forces behind species dynamics/changes include primary production, temperature and fishing pressure (Macias et al., 2015; Piroddi et al., 2017; Agnetta et al., 2019; Moullec et al., 2019). Recent studies (Piroddi et al., 2015, 2017), which coupled a food web model with an hydrodynamic-biogeochemical model, have highlighted the important role and impact of the environment and anthropogenic pressures (e.g., fishing pressure) in shaping the dynamics of the Mediterranean marine resources. In particular, it has been shown that such drivers have explained historical trends (1950–2011) of several groups of the food web (from low to high trophic levels), highlighting a reduction of important forage and demersal fish species (respectively ~40% and ~17%), marine mammals (~41%), and increases of the organisms at the bottom of the food web (~23%, invertebrates) (Fig. 3).

**Fig. 4:**
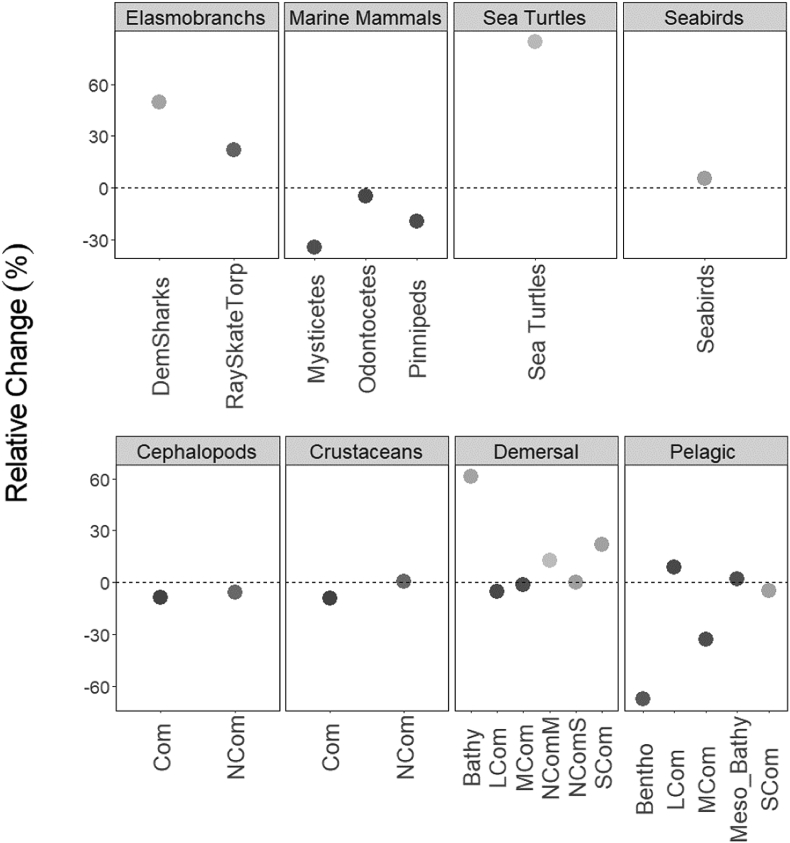
Modelled relative change (%) of biomass for the main functional groups of the Mediterranean Sea LME. The relative change was calculated as ((*End*-*Base*)/*Base*)*100) where *End* is the end period (2012–2016) and *Base* the baseline period (1995–2000, dashed line). DemSharks: demersal sharks; RaySkateTorp: Rays, Skates and Torpedoes; Com: commercial; NCom: non-commercial; Bathy: bathydemersal fish; L: large, M: medium and S: small; Bentho: benthopelagic fish; Meso_Bathy: meso and bathypelagic fish.

Using the same modelling approach to investigate more recent years (1995–2016) and more functional groups, the picture highlights shifts in the dynamics of the Mediterranean food web. Current biomass of marine mammals, cephalopods (both commercial and non-commercial), commercially important crustaceans, commercially important fish as large and medium demersals, medium and small pelagics and benthopelagics show a decrease compared to 1990s. The biomass of sea turtles, seabirds, elasmobranchs, commercial large pelagics (e.g., tunas), commercial small demersals (e.g., Mullidae) and non-commercial fish like bathydemersals, medium demersals and meso and bathypelagics indicate an increase. As for the rest of the functional groups, model suggests a slight change of less than ±0.5% compared to 1990s (Fig. 4).

When looking at forecast scenarios, a recent analysis conducted by Moullec et al. (2019), which coupled together a climate, biogeochemical and multispecies dynamic model, showed the response of the Med-LME to future scenarios of climate change (high emission scenario RCP8.5) (IPCC, 2014) and fishing mortality (kept as observed in recent years). The results concluded that, between 2020 and 2100, increase in temperature and salinity will increment the total biomass and catch (2–15% and 0.3–5% respectively) of fish, mainly of pelagic thermophilic and/or exotic species having smaller sizes and low trophic level (e.g., sardines and anchovies).

### 1.2 Status of commercial stocks and elasmobranchs at GSA level

Regarding the status of the Med-LME stocks for which we have available and validated assessments (FAO, 2018a), the overall picture shows that most of the Med-LME stocks continue to be fished outside biologically sustainable limits. Recent independent assessments (Froese et al., 2018; Hilborn et al., 2020) confirm the bad status of Med-LME due to long-lasting and ongoing overexploitation that has been previously reported (Colloca et al., 2013; Vasilakopoulos et al., 2014; Tsikliras et al., 2015).

**Fig. 5:**
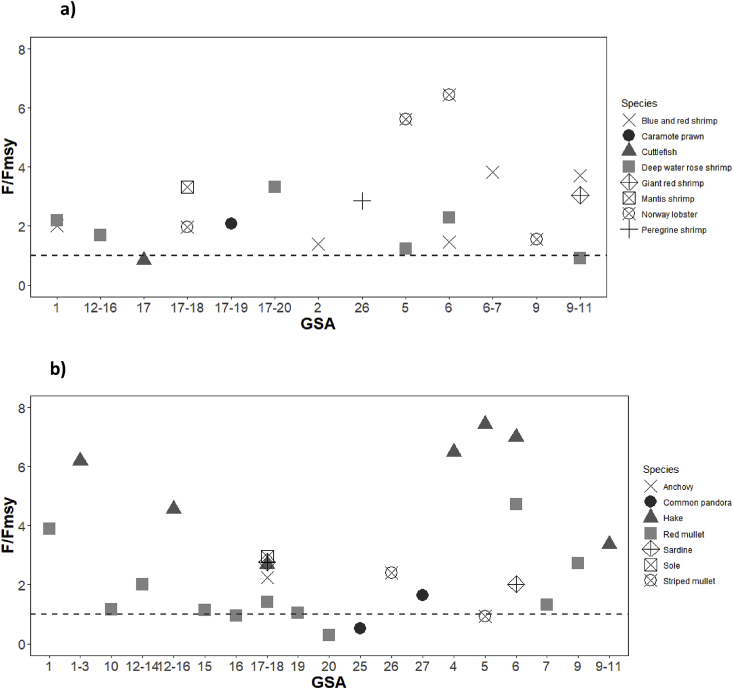
Summary of the most recent F/F_MSY_ ratios available for Med-LME commercial stocks in the period 2017–2018: a) crustaceans and cephalopods; b) fish. The dotted line indicates sustainable fishing mortality: F/F_MSY_ = 1. (Source: GFCM, 2018a, GFCM, 2018b; STECF, 2019).

Data from more than 80 stocks of fish and crustaceans assessed in the period 2002–2014 showed that, for about 90% of the stocks, the current fishing mortality (F) was higher than the fishing mortality at MSY (F_MSY_) (Colloca et al., 2017a). The overfishing pattern has not improved recently according to the last available assessments. In the period 2017–2018, only 6 stocks (12%) of over 48 assessed resulted as sustainably exploited (GFCM, 2018a, GFCM, 2018b; STECF, 2019). These included red mullet (*Mullus barbatus*) in South of Sicily (GSA 16) and Eastern Ionian Sea (GSA 20); striped red mullet (M*ullus surmuletus*) in the Balearic Islands (GSA 5), common pandora (*Pagellus erythrinus*) in Cyprus (GSA 25), deep water rose shrimp (*Parapenaeus longirostris)*, and finally cuttlefish (*Sepia officinalis*) in the Adriatic Sea (GSA 17) (Fig. 5).

The highest overfishing (F/F_MSY_»1) was found for hake (*Merluccius merluccius*) either in western or central Med-LME with current fishing mortality (F) 3–8 times higher than F at maximum sustainable yield (F_MSY_). There is still uncertainty in the stock status of the main forage fish species, anchovy (*Engraulis encrasicolous*) and sardine (*Sardina pilchardus*), although the few formally accepted assessments (i.e sardine and anchovy in North Adriatic, sardine in North Spanish coasts) have shown an overexploitation status and a generally low stock biomass. Several species of crustaceans have increased their commercial importance in the last years, including non-indigenous species such as the peregrine shrimp (*Penaeus stebbingi*) in Egypt.

A peculiar case is the deep-water rose shrimp (*Parapenaeus longirostris*) whose stocks are expanding northward as an effect of global warming and its associated warming water temperatures (Colloca et al., 2014). The landings increased in the last years and the stock appeared as sustainably exploited along the western coast of Italy (GSAs 9–11). The other crustacean stocks assessed were found generally to be overfished (Fig. 5b), with Norway lobster (*Nephrops norvegicus*) exploited far from sustainability in North Spain and Balearic Islands (GSA 6 and 5). Similarly, the deep water trawl fishery has exploited beyond F_MSY_ the blue and red shrimp (*Aristeus antennatus*) on the continental slope of North Spain (GSA 6) and North Tyrrhenian and Ligurian Sea (GSA 9) and the giant red shrimp (*Aristaeomorpha foliacea*) in this latter area. The only cephalopod species assessed was cuttlefish (*Sepia officinalis*) in North Adriatic (GSA 17) where it appeared as sustainably exploited in the last years.

Overfishing of commercial stocks has also led to population decreases of common by-catch species including elasmobranchs. These are commonly caught as by-catch, for example, of pelagic fisheries targeting large pelagic species such as bluefin tuna and swordfish. There is a growing body of evidence about the declining trend of populations of several species of both sharks and batoids during the last 50 years in different parts of the Mediterranean Sea (Ferretti et al., 2008; Maynou et al., 2011; Guijarro et al., 2012; Barausse et al., 2014; Colloca et al., 2017b). This was and still is likely due the effect of increasing trends in fishing effort (Garcia, 2011) and low population resilience to harvesting, so that the Mediterranean region is now depicted as the global area with the highest proportion of threatened species (with 40% of the species), classified as critically endangered, endangered or vulnerable (Dulvy et al., 2014, 2016). Yet, illegal and underreporting catches, a general aggregation in catch statistics and limited data on their abundance at regional scale still limit their overall assessment (Cashion et al., 2019; Giovos et al., 2020); thus, it is not surprising that there are inconsistencies between models outputs on their status (see above section on food web).

### 1.3 Regional catch trends

Catch (equivalent to landings for the purposes of this study) has been widely used to assess the exploitation pattern of many stocks globally when no other data are available (Pauly et al., 2013). Novel methods of assessing stock status have been designed to use only catch and resilience in the absence of abundance or biomass data (Froese et al., 2017; Demirel et al., 2020). In addition, surplus production models are particularly useful in data poor fisheries and have been extensively used to assess the status of data-poor stocks throughout the world, including the Med-LME (Froese et al., 2018).

With a constant fishing effort and accounting for technological advancements that improve catchability (effort creep), which increases the overall effectiveness of fishing (Palomares and Pauly, 2019), the temporal variability of catch is a good indicator of exploitation and fisheries sustainability (Pauly et al., 2013). However, detailed assessments are required for all commercial stocks to quantify the exploitation pattern and stock status in terms of biomass.

In this study, an updated (1970–2017) time series of reported catch is presented per sub-regional division (Fig. 1) based on the latest available information of the official data (FAO, 2020). Highly migratory species (e.g. tuna, swordfish) that are being exploited and reported across the Med-LME were excluded from the analyses. Reported catches from Greece (Ionian Sea in central Mediterranean and Aegean Sea in the eastern Mediterranean) for 2016 and 2017 were corrected downwards to account for presentist bias (Tsikliras et al., 2020). It should be noted here that, although fishing is the main driving force of exploited marine populations, climate and environmental forcing may also play an important role especially for pelagic fishes (Tsikliras et al., 2019).

**Fig. 6:**
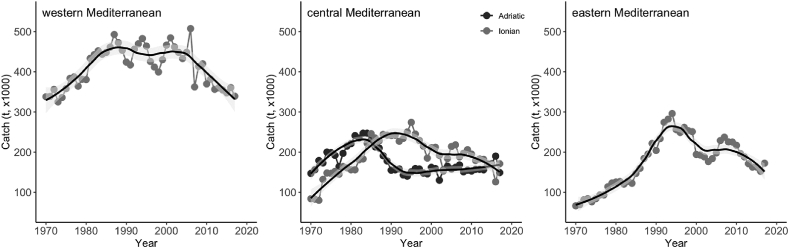
Total reported catch (thousand tonnes) in the western, central (Adriatic and Ionian Sea) and eastern Med-LME for the period 1970–2017 (updated from Tsikliras et al., 2015). A smoothing line with confidence limits was added in the time series to show the overall reported catch trend in each area.

In the western Med-LME, total reported catch was rather stable for about 25 years (1980–2005) and constantly declined thereafter down to 66% of the highest catch (Fig. 6). In the Adriatic Sea (northern part of central Med-LME), total reported catch increased from 1970 to 1984, then declined rapidly by mid 1990s and stabilized at around 70% of the highest value (Fig. 6). In the Ionian Sea (southern part of central Mediterranean), total reported catch increased from 1970 to 1994, then smoothly and continuously at around 60% of the highest value in 2017. Finally, in the eastern Mediterranean the reported catch trend is similar to that of the central area with an increase in catch from 1970 to 1994 and a decline thereafter to around 65% of maximum catch in 2017 (Fig. 6). The smooth declining trend in the eastern subarea is interrupted by lower catch values from 2000 to 2005.

### 1.4 Med-LME food webs are shrinking

**Fig. 7:**
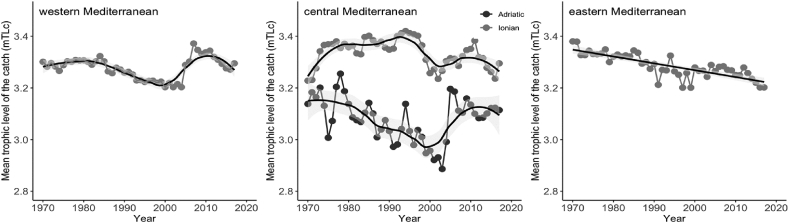
Mean weighted trophic level of the reported catch (mTLc) in the western, central (Adriatic and Ionian Sea) and eastern Med-LME for the period 1970–2017 (updated from Tsikliras et al., 2015). A smoothing line with confidence limits was added in the time series to show the overall mTLc trend in each area.

**Fig. 8:**
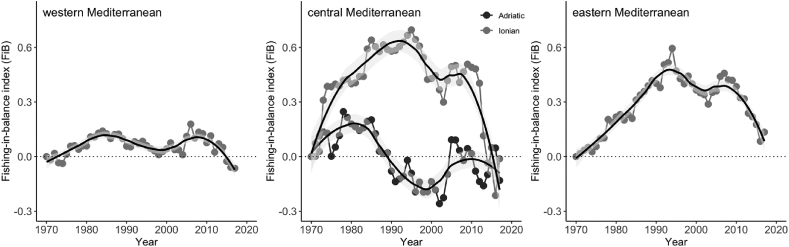
Fishing-in-balance (FiB) index in the western, central (Adriatic and Ionian Sea) and eastern Med-LME for the period 1970–2017 (updated from Tsikliras et al., 2015). A smoothing line with confidence limits was added in the time series to show the overall FiB trend in each area.

The mean weighted trophic level of the catch (mTLc) has been widely used to examine the selective removal of larger organisms and individuals by fishing (Pauly et al., 1998) and the gradual decline in size both within and among species, which results in shrinking marine food webs (Stergiou and Tsikliras, 2011). The fishing-in-balance (FiB) is used to examine the effect of fishing on marine ecosystems with increasing or decreasing FiB values indicating a geographic expansion or contraction (or collapse) of the fishery in concern, respectively (Pauly et al., 2000). FiB attains a value of 0 for the first year of the series and does not vary in periods in which trophic level and catches change in opposite directions. The updated mTLc and FiB are presented here for the period 1970–2017 (Fig. 7, Fig. 8) using the exact same methodology and stocks with a previous publication that referred to the period 1970–2010 (Tsikliras et al., 2015).

In the western Med-LME (1970–2018), mTLc declined from around 3.30 (1970–1985) to around 3.20 (1986–2004), at a rate of 0.03 per decade (Fig. 7). After an upward shift to around 3.37 between 2005 and 2007, owing to the collapse of anchovy (*Engraulis encrasicolus*) and sardine (*Sardina pilchardus*) fisheries in the Gulf of Lions (Saraux et al., 2019), mTLc started to decline again (Fig. 7). The FiB index declined from 1984 to 2004, shifted to its maximum value in 2006 and since then it declined steadily to its lowest value in 2017 (Fig. 8).

In the Adriatic Sea mTLc followed a similar pattern with the western Med-LME as it declined from around 3.20 in early 1970s down to 2.89 in 2003. After an upward shift to 3.20 in 2005, mTLc started to decline again down to 3.11 in 2017 (Fig. 7). Given the long stability in catch after 1990, the fluctuation of FiB mostly followed the mTLc variability (Fig. 8). In the Ionian Sea, mTLc increased to 3.40 in the mid 1990s and then declined to around 3.30 after 2015 (Fig. 7). Following the constant decline of the catch and the decline of mTLc after the mid 1990s, the trend of FiB has been declining since 1995 (Fig. 8).

The trend of mTLc in the eastern Med-LME is clear and consistent exhibiting a constant decline throughout the time series (Fig. 7). The rate of mTLc decline is around 0.03 per decade, from 3.38 in 1970 to 3.21 in 2017 (Fig. 7). FiB followed a similar trend with the catch and rapidly increased from 1970 to 1994 to its highest value in 1994 and then declined to around 30% of its maximum value in 2017 (Fig. 8).

The declining trends of mTLc and FiB across areas that are more pronounced and longer lasting in the eastern Med-LME and more recent in the central and western areas are clear indicators of contracted fisheries and of shrinking marine food webs. Recent large-scale stock assessments (Froese et al., 2018) and ecosystem models (Piroddi et al., 2017) concluded that biomass and reported catch declines are the result of overexploitation and that these trends could be reversed with less fishing in time and space.

### 1.5 Management and the move forward

Several regional/international organisations, agreements and initiatives are involved in the protection of the Med-LME marine biodiversity and in the maintenance of a sustainable economic development. Most notably, the Convention for the Protection of the Marine Environment and the Coastal Region of the Mediterranean (Barcelona Convention) – which includes the United Nations Environment Programme (UNEP)'s Mediterranean Action Plan (MAP) -, the Agreement on the Conservation of Cetaceans in the Black Sea, Mediterranean Sea and contiguous Atlantic area (ACCOBAMS), the Food and Agriculture Organization (FAO) with several sectoral agreements and initiatives - such as the FAO Compliance Agreement, the General Fisheries Commission for the Mediterranean (GFCM) and the International Commission for the Conservation of Atlantic Tunas (ICCAT) – and the Convention on Biological Diversity (CBD).

Within Europe, three main legislations exist to preserve and sustainably use the Mediterranean Sea marine resources: the Common Fishery Policy (CFP, Regulation (EU) No 1380/2013), which aims at ensuring that fishing is environmentally, economically and socially sustainable; the Marine Strategy Framework Directive (MSFD, 2008/56/EC), which aims to achieve or maintain Good Environmental Status (GES) by 2020; and the EU Biodiversity Strategy to 2030 (COM (2020) 380) which aims to halt the loss of biodiversity and ecosystem services. Recently, under the CFP, a first multiannual plan for fisheries exploiting demersal stocks in the western Mediterranean Sea has been enforced by the European Union (Regulation (EU) 2019/1022 of the European Parliament and of the Council of June 20, 2019 amending Regulation (EU) No 508/2014), with strict spatial and temporal regulations and restrictions, to avert the overexploitation of marine resources that has lead to bad stock status in the area.

Despite the presence of such frameworks, agreements and initiatives, the Med-LME still is one of the most impacted regions of the world (Costello et al., 2010) with unsustainable fishing pressure and overexploitation of many stocks (FAO, 2018b), increase in invasive species (Katsanevakis et al., 2014), in plastic/litter pollution (Cózar et al., 2015; Macias et al., 2019) and extreme climate events (Verdura et al., 2019). Major causes behind this degradation are related to the socio-political complexity of the region (21 countries from Europe, Asia and Africa with different political and cultural systems), the inadequacies of national management plans and, often, to the non-observance of scientific advices (Cardinale et al., 2017). In relation to fisheries, one of the main issues is that most policy measures and European regulations are designed and developed for single species fishery (e.g. the landing obligation) and do not consider the mixed nature of the Mediterranean fisheries (and the dynamics among the stocks). Mixed and multispecies fisheries result in large numbers (and quantities) of by-catch species for which data are scarce and their status is not being routinely evaluated (Froese et al., 2020).

Because fisheries and other anthropogenic pressures are expanding at fast pace in the Med-LME, the move towards a holistic approach to ecosystem marine management in the basin seems to be necessary. Ecological and socio-ecological modelling tools, including stock assessments, have been recognized to be essential in addressing this issue (Hyder et al., 2015; Heymans et al., 2018). These models have long been used and developed in academic and research settings but only few examples exist of their use in an ensemble framework to directly support policies and management decisions (Rosenberg et al., 2018).

Currently, for example, ecosystem models (Piroddi et al., 2011; Dimarchopoulou et al., 2019) and stock assessments (Froese et al., 2018), developed for different areas of the Med-LME, predict that true effort reductions (i.e. accounting for effort creep and hauling time) through confinement of fleet numbers and days at sea should be accompanied by permanent spatial restrictions of trawling on essential fish habitats (nursery and spawning area), while the coastal zone should be reserved for small-scale coastal vessels and selective fisheries (nets, longlines) (Dimarchopoulou et al., 2018).

Two global disasters (World Wars I and II) were the first two involuntary “experiments” on the positive effects of less fishing on marine populations and ecosystems (D’Ancona, 1926; Russell, 1942; Graham, 1949; Beverton, 1957; Thurstan et al., 2010). Unfortunately, a third one (the covid-19 disease) is ongoing in 2020 and has so far caused a drastic decline in fishing effort (Global Fishing Watch, 2020). The precautionary quarantine and the lockdown caused a suspension of fishing activities (including recreational fishing) in many areas and has also decreased the demand for fish as the markets are not operating as usual. Especially in the Med-LME, fishing has been suspended for at least March and April 2020 and touristic activities are minimized. In the following years, a rebuilding of stock biomass and population structure for short-lived species is expected only to prove that effort declines will rebuild stocks. Today, fisheries managers have all the necessary tools to do the job of sustainably exploiting marine populations; no need to rely on pandemics and wars for stocks to recover.

## Declaration of competing interest

The authors declare that they have no known competing financial interests or personal relationships that could have appeared to influence the work reported in this paper.
